# EmoTweetID: A dataset of Indonesian tweets for emotion classification and word embedding construction

**DOI:** 10.1016/j.dib.2026.113119

**Published:** 2026-07-31

**Authors:** Kuncahyo Setyo Nugroho, Fitra Abdurrachman Bachtiar, Wayan Firdaus Mahmudy, Matthew Martianus Henry, Mahmud Isnan, Gusti Pangestu, Bens Pardamean

**Affiliations:** a Bioinformatics and Data Science Research Center, Bina Nusantara University, Jakarta, 11530, Indonesia; b Informatics Engineering Department, Faculty of Computer Science, Universitas Brawijaya, Malang, 65145, Indonesia; c Computer Science Department, School of Computer Science, Bina Nusantara University, Jakarta, 11530, Indonesia; d Computer Science Department, BINUS Graduate Program – Master of Computer Science, Bina Nusantara University, Jakarta, 11530, Indonesia

**Keywords:** Indonesian tweets, Natural language processing, Text processing, Text mining, Emotion classification, Social media analysis

## Abstract

The EmoTweetID dataset is a large-scale open resource of Indonesian tweets curated for emotion classification and word embedding, addressing the scarcity of publicly available datasets for this low-resource language. Tweets were collected from the X platform (formerly Twitter) using the snscrape library, yielding >4.5 million raw tweets based on basic and derived emotion keywords from Ekman’s six basic emotions. After cleaning to remove duplicates, irrelevant tweets, and sensitive elements, a corpus of 3,126,987 clean unlabeled tweets was compiled from the first scraping stage. The second stage gathered 2,243 clean tweets annotated through lexicon-based and manual methods, into six emotion classes based on Ekman’s basic emotions: anger, disgust, fear, joy, sadness, and surprise. The manual annotation by three psychology students used majority voting and achieved substantial inter-annotator agreement, with a Fleiss’ Kappa score of 0.7323. Two pretrained embeddings, Word2Vec and fastText, were trained on the corpus using a 300-dimensional skip-gram architecture to enrich semantic representation. Baseline evaluations using BiLSTM with fastText achieved a weighted F1-score of 0.8285 on the human-annotated set, demonstrating the practical utility of the dataset and embeddings for the downstream emotion classification task. Publicly available on Mendeley Data, the EmoTweetID dataset provides a valuable foundation for advancing Indonesian natural language processing by enabling unsupervised pre-training and supervised multi-class emotion classification.

Specifications Table

**Table:** 

Subject	Computer Sciences
Specific subject area	Natural Language Processing, Affective Computing
Type of data	Text (CSV-formatted) Word Embedding Model
Data collection	The data were collected from X (formerly Twitter) using the snscrape library version 0.2.0 on Python 3.8 in the Google Colab environment. The parameter lang='id’ was applied to ensure that only Indonesian tweets were retrieved. The data collection was conducted during two periods: January 1–December 31, 2020 and January 1–31, 2021. The data collection process used basic emotion keywords from Ekman and their derived emotional expressions. In total, more than >4.5 million raw tweets were collected. After the data cleaning, 3,126,987 unlabeled tweets were compiled for the corpus, and 2,243 tweets were prepared for annotation.
Data source location	Institution: The tweets were collected at Universitas Brawijaya, subsequently processed and analyzed at Bina Nusantara University Country: Indonesia
Data accessibility	Repository name: Mendeley Data Data identification number: 10.17632/jzgnjsff9f.5 Direct URL to data: https://data.mendeley.com/datasets/jzgnjsff9f/5 Instructions for accessing these data: The dataset is publicly available for research purposes. No login is required to download.
Related research article	None

## 1 Value of the Data

• EmoTweetID [1] consists of Indonesian tweets collected from X (formerly Twitter) using explicit emotion-related keywords. The dataset comprises three main files: (1) EmoTweetID-Corpus.csv consists of 3,126,987 unlabeled tweets, (2) EmoTweetID-Lexicon.csv consists of 2,243 tweets automatically annotated using the Indonesian NRC Emotion Lexicon (EmoLex) [2], and (3) EmoTweetID-Human.csv consists of 2,243 tweets manually annotated by three psychology students.
• The unlabeled corpus (EmoTweetID-Corpus.csv) is suitable for unsupervised tasks such as language model pre-training and semantic exploration of emotion-related expressions. Meanwhile, the annotated sets (EmoTweetID-Human.csv and EmoTweetID-Lexicon.csv) are suitable for supervised tasks, particularly multi-class classification based on Ekman’s six basic emotions: anger, disgust, fear, joy, sadness, and surprise [3].
• This dataset provides valuable resources for affective computing research in limited-resource languages such as Indonesian, especially for modeling emotional expressions in short and informal texts commonly found on social media. It supports the development of context-aware and empathetic natural language processing applications, including emotion-aware dialogue systems and recommendation systems.
• Beyond affective computing research, the dataset also provides valuable material for studies in computational social science, digital psychology, and linguistics. It can be a benchmark for future Indonesian emotion recognition and social media language understanding research.
• The EmoTweetID dataset has potential applications across multiple domains, including public health monitoring, where it can track population-level emotional trends, and customer service systems, where it can support the development of emotion-aware chatbots.
• This repository also contains two pretrained word embedding models, TweetID-Word2Vec.zip and TweetID-fastText.zip. These embeddings were trained on EmoTweetID-Corpus.csv and can perform as feature extractors or embedding initializers for downstream Indonesian NLP tasks, particularly for social media text analysis.
• The dataset follows the FAIR Data Principles [4]: Findable via DOI, Accessible publicly through Mendeley Data, Interoperable in CSV and Gensim-compatible formats, and Reusable under an open license.

## 2 Background

Emotion modeling in Indonesian text remains underdeveloped due to the scarcity of high-quality publicly available datasets. Unlike sentiment analysis, emotion classification requires more specific and context-dependent labels and must be grounded in psychological theories of emotion. One widely adopted theory is Ekman’s model, which defines six basic emotions: anger, disgust, fear, joy, sadness, and surprise [3]. Recent advances in emotion recognition across modalities, including facial and textual signals, further highlight the importance of linguistically grounded resources for understanding emotions [5].

To address this gap, the EmoTweetID dataset was developed to advance research and applications in natural language processing (NLP) and affective computing for the Indonesian language. The data were collected from X (formerly Twitter) using emotion keyword-based queries and arranged into three parts: (1) unlabeled tweets for the corpus, (2) automatically annotated tweets using the Indonesian NRC Emotion Lexicon (EmoLex), and (3) manually annotated tweets by three psychology students.

This dataset was designed with two primary objectives: (1) to provide large-scale social media data for pre-training Indonesian language models, and (2) to establish a benchmark for multi-class emotion classification tasks. This paper describes the dataset creation process and provides open access to all dataset versions to ensure transparency, reproducibility, and future research and development.

## 3 Data Description

The EmoTweetID dataset consists of five publicly available files on the Mendeley Data repository [1], categorized into three main groups: an unlabeled tweet corpus, annotated tweets, and pretrained word-embedding models. All CSV files are encoded using UTF-8 to ensure compatibility and proper representation of Indonesian characters. Details of each file are as follows.

The first file, EmoTweetID-Corpus.csv, contains 3,126,987 unlabeled tweets with a single column named *tweet*. This file contains raw textual data with no additional labeling or pre-processing beyond basic text cleaning.

The second and third files, EmoTweetID-Lexicon.csv and EmoTweetID-Human.csv, contain the same 2,243 tweets annotated using two different methods. EmoTweetID-Lexicon.csv was automatically annotated using the Indonesian NRC Emotion Lexicon (EmoLex), while three psychology students manually annotated EmoTweetID-Human.csv. Both files include two columns: *tweet* and *label*, where the label is one of Ekman’s six basic emotions: anger, disgust, fear, joy, sadness, or surprise. The emotion label distributions in both files are presented in Table 1. In addition, Table 2 provides descriptive statistics of the tweet lengths, measured in characters and tokens. No missing or incomplete tweets are included in any of the released files. Tweets that resulted in empty content after cleaning are removed prior to dataset construction.

**Table 1:** The tweet count distribution for each emotion label is based on two annotation methods: automatic annotation using the Indonesian NRC EmoLex and manual annotation, both calculated from 2,243 tweets in the EmoTweetID-Lexicon.csv and EmoTweetID-Human.csv files.

	Anger	Joy	Fear	Disgust	Sadness	Surprise
NRC EmoLex	422	547	360	305	314	295
Human annotation	475	429	395	355	303	286

**Table 2:** Descriptive statistics of tweet length in characters (Char Length) and tokens (Token Length), calculated from 2,243 tweets in both EmoTweetID-Human.csv and EmoTweetID-Lexicon.csv.

	Mean	Std	Min	25 %	50 %	75 %	Max
Char Length	103.23	77.86	5.00	40.00	79.00	147.50	284.00
Token Length	19.67	15.03	1.00	8.00	15.00	28.00	75.00

The remaining two files, TweetID-Word2Vec.zip and TweetID-fastText.zip, are pretrained word embedding models trained on EmoTweetID-Corpus.csv using Word2Vec [6] and fastText [7]. Each ZIP file contains two files (.npy and .wordvectors) which are compatible with Gensim version 3.6.0 or later.

To ensure user privacy, no timestamp fields are included in any of the released files, and temporal information from the original tweets has been fully removed. For improved clarity and usability, Table 3 summarizes all files included in the Mendeley Data repository, including file size, number of rows, number of columns, and brief descriptions of their contents. Note that the pretrained word embedding files follow a distinct naming convention (TweetID-) to differentiate them from the corpus and annotated tweet files (EmoTweetID-), reflecting their different roles within the dataset.

**Table 3:** Overview of all files provided in the EmoTweetID dataset repository.

File name	Size	Rows	Columns	Description
EmoTweetID-Corpus.csv	208 MB	3,126,987	1	Unlabeled Indonesian tweets
EmoTweetID-Human.csv	254 KB	2,243	2	Emotion-labeled tweets manually annotated
EmoTweetID-Lexicon.csv	256 KB	2,243	2	Emotion-labeled Indonesian tweets using NRC EmoLex
TweetID-Word2Vec.zip	169 MB	-	-	Pretrained Word2Vec
TweetID-fastText.zip	882 MB	-	-	Pretrained fastText

## 4 Experimental Design, Materials, and Methods

**Fig. 1:**
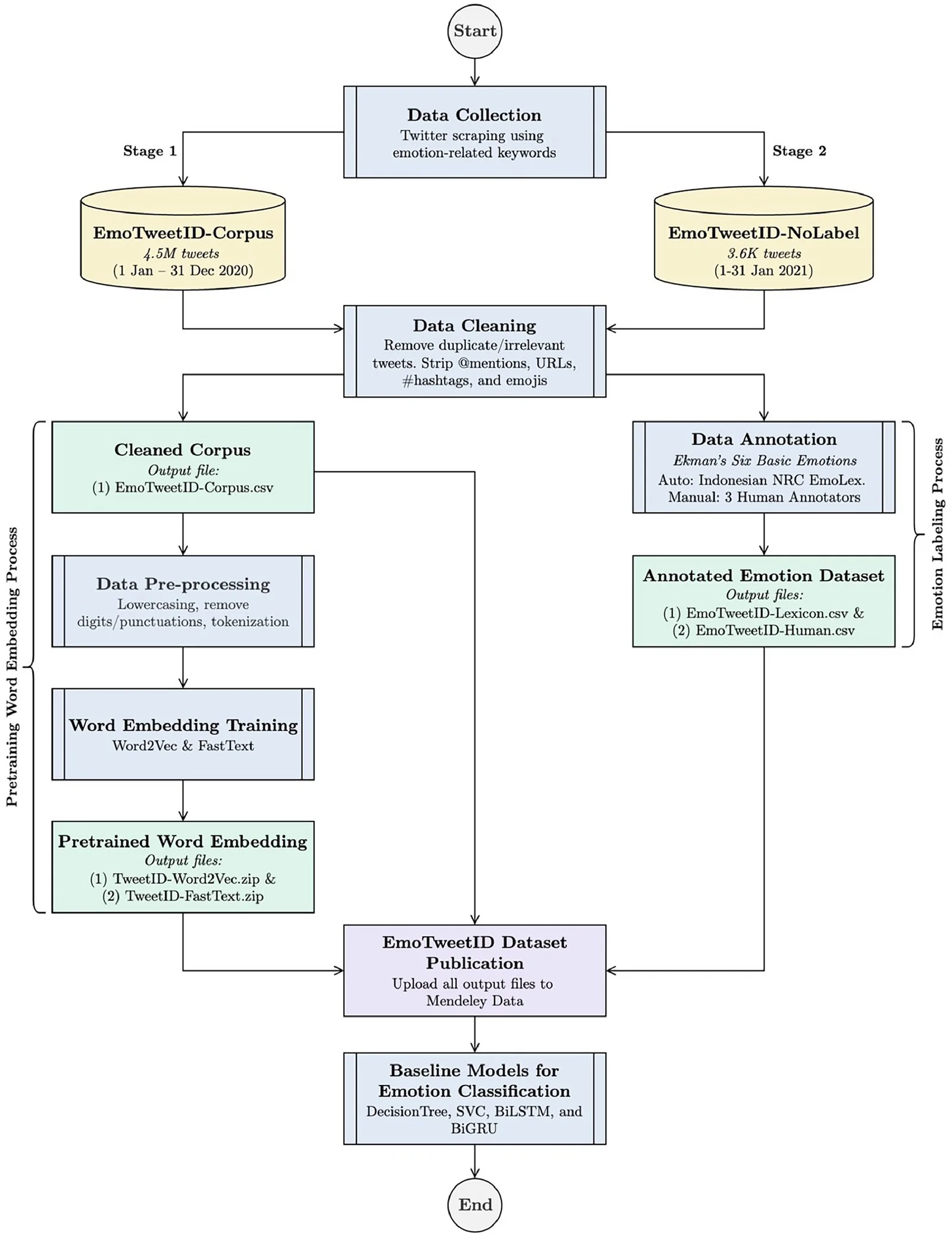
Workflow of the EmoTweetID dataset construction.

The workflow for constructing and evaluating the EmoTweetID dataset is illustrated in Fig. 1. The explanation of each process is described in the following sections.

### 4.1 Data collection

The data were collected from the X platform (formerly Twitter) using the snscrape library (version 0.2.0) [8], executed in Python 3.8 within a Google Colab environment. The parameter lang='id’ was applied to ensure that only Indonesian-language tweets were retrieved. Data collection was conducted in two stages to construct the EmoTweetID dataset.

The first stage targeted tweets posted between January 1 and December 31, 2020, aiming to collect 4.5 million tweets. Six emotion keywords were used, corresponding to Ekman’s basic emotions in Indonesian: *marah* (anger), *jijik* (disgust), *takut* (fear), *senang* (joy), *sedih* (sadness), and *terkejut* (surprise), as shown in the left column of Table 4. Each keyword was targeted to collect 750,000 tweets. In the second stage, scraping was performed within a narrower time window, from January 1 to 31, 2021, to collect 3,600 tweets. This phase used the basic emotion keywords and their derived emotional expressions, as shown in the right column of Table 4. Each derived emotion keyword was targeted to retrieve 150 tweets.

**Table 4:** List of basic emotion keywords and their derived expressions used in the scraping process.

Basic emotion keywords	Derived expression keywords
*Marah* (anger)	*Kesal* (annoyed), *murka* (furious), *benci* (hateful)
*Jijik* (disgust)	*Muak* (nauseated), *risih* (uncomfortable), *penat* (tired)
*Takut* (fear)	*Ngeri* (horrified), *cemas* (anxious), *gugup* (nervous)
*Senang* (joy)	*Bangga* (proud), *bahagia* (happy), *puas* (satisfied)
*Sedih* (sadness)	*Kecewa* (disappointed), *duka* (grief), *sengsara* (miserable)
*Terkejut* (surprise)	*Heran* (amazed), *terpesona* (awestruck), *tertipu* (deceived)

All scraped tweets from both stages were stored in CSV format to facilitate the annotation process and subsequent model training. The full data collection script, including scraper configuration and query parameters, is publicly available on GitHub repository [9].

### 4.2 Data cleaning

The scraped tweets were collected in raw form and thus contained irrelevant information and potential personal data. Therefore, a data cleaning process was applied before further use. The raw tweets were not included in the public EmoTweetID repository for ethical and privacy reasons.

The data cleaning process consisted of several steps. First, all duplicate tweets were removed to avoid redundancy. Second, irrelevant tweets, such as spam, promotional content, or posts without emotional content, were eliminated through manual review by three psychology students based on their expert judgment in identifying emotionally relevant tweets. Third, unnecessary elements for analysis, including user mentions (@username), URLs (e.g., ht-tps://, ht-tp://, or w-ww.), and hashtags (#) were removed using the tweet-preprocessor library (version 0.6.0) [10]. Fourth, emojis and emoticons such as  or :-) were converted into their Indonesian text equivalents using an emoji-to-text mapping dictionary comprising 1,513 entries, to make them processable by text-based models [9]. An example of the data cleaning result is shown in Table 5, which illustrates the transformation from raw to cleaned tweets.

**Table 5:** Examples of transformation from raw to clean tweets during the data cleaning process.

Raw tweet	Clean tweet
@smileyhob *Dengan senang hati mull*https://t.co/Ap5l9V2FKM **English translation:** @smileyhob wholeheartedly mull https://t.co/Ap5l9V2FKM	*Dengan senang hati mull cinta* **English translation:** Wholeheartedly mull love
@detikcom *ada yg salah dengan negara kita, makin banyak orang yg halu dan gak percaya dengan pemerintahan, harusnya jadi perhatian pemerintah nih, bisa jadi krn mereka kecewa dgn pemerintah dan merasa ada harapan baru.. Krn yg ikut banyak lho.. Lama2 bisa jadi gerakan revolusi lho ini..* #*KECEWA* **English translation:** @detikcom something is wrong with our country, more and more people are delusional and no longer trust the government, it should be the government's concern, maybe because they are disappointed with the government and feel there is new hope.. Because many people are joining.. Eventually this could become a revolutionary movement.. #DISAPPOINTED	*ada yg salah dengan negara kita, makin banyak orang yg halu dan gak percaya dengan pemerintahan, harusnya jadi perhatian pemerintah nih, bisa jadi krn mereka kecewa dgn pemerintah dan merasa ada harapan baru.. Krn yg ikut banyak lho.. Lama2 bisa jadi gerakan revolusi lho ini.. KECEWA* **English translation:** something is wrong with our country, more and more people are delusional and no longer trust the government, it should be the government's concern, maybe because they are disappointed with the government and feel there is new hope.. Because many people are joining.. Eventually this could become a revolutionary movement.. DISAPPOINTED

After completing the entire data cleaning process, a total of 3,126,987 unlabeled cleaned tweets were stored in EmoTweetID-Corpus.csv, and 2,243 cleaned tweets were prepared for annotation in EmoTweetID-Lexicon.csv and EmoTweetID-Human.csv. The complete data cleaning script, including pre-processing functions and configuration settings, is available in the GitHub repository [9].

### 4.3 Data pre-processing

Data pre-processing was applied only to EmoTweetID-Corpus.csv to prepare the data before word embedding training. The process was not applied to EmoTweetID-Lexicon.csv and EmoTweetID-Human.csv, as both files were intended for annotation and baseline model evaluation.

The first pre-processing step was to convert all tweet characters to lowercase. Next, all digits and punctuation marks were removed. The final step was tokenization, which transformed each tweet into smaller units (tokens). To train Word2Vec, tokenization was performed at the word level. Meanwhile, tokenization was performed at the subword level for fastText training.

### 4.4 Data annotation

Data annotation aimed to assign an emotion category to each tweet in EmoTweetID-Lexicon.csv and EmoTweetID-Human.csv based on Ekman’s six basic emotions: anger, disgust, fear, joy, sadness, and surprise. The annotation process used two methods: lexicon-based and manual annotation by human annotators.

Lexicon-based annotation was performed automatically using the Indonesian NRC Emotion Lexicon (EmoLex) on EmoTweetID-Lexicon.csv. The EmoLex dictionary is structured as JSON format, mapping each word to one or more emotion categories, as shown in Table 6. Each tweet was tokenized into words, then each token was checked against the EmoLex dictionary. The corresponding emotion score was incremented if a token was found in the dictionary. The final emotion label for each tweet was determined based on the highest emotion score. In cases where two or more emotions received equal scores, the first emotion encountered in the scoring dictionary was assigned as the final label. The pseudocode for the lexicon-based annotation procedure is presented in Table 7. Since all tweets are collected using emotion-specific keywords that are present in the EmoLex dictionary, each tweet inherently contains at least one matching lexicon entry, thus no tweet receives an all-zero score and all tweets are assigned one of Ekman’s six, with no neutral label produced.

**Table 6:** Sample entries from the Indonesian NRC EmoLex Dictionary.

1 2 3 4 5 6 7 8 9 10 11 12 13 14 15 16 17	"anarki":[ "anger", "fear" ], "anarkis":[ "anger", "fear" ], "anarkisme":[ "anger", "fear" ], "ancaman":[ "anger", "fear" ],

**Table 7:** Pseudocode for lexicon-based emotion annotation.

1 2 3 4 5 6 7 8 9 10 11 12 13 14 15 16 17 18 19	**procedure**AnnotateLexicon(tweets, emolex): labels←[] scores_all←[] **for each**tweet*in*tweets**do** tokens←*tokenize*(tweet) scores←{anger:0, disgust:0, fear:0, joy:0, sadness:0, surprise:0} **for each**token*in*tokens**do** **if**token*in*emolex**then** **for each**emotion*in*emolex[token]**do** scores[emotion]←scores[emotion] + 1 **end for** **end if** **end for** label←*argmax*(scores) labels.*append*(label) scores_all.*append*(scores) **end for** **return**labels, scores_all **end procedure**

In addition, three psychology students manually annotated the dominant implied emotion in each tweet on EmoTweetID-Human.csv. The final emotion label for each tweet was determined through majority voting among the three annotators. In cases where all three annotators assigned different labels, a designated annotator conducted a final review to determine the conclusive label. Cohen’s Kappa was calculated for each pair of annotators to evaluate inter-annotator consistency, and Fleiss’ Kappa was calculated for all annotators collectively, as shown in Table 8. The Kappa score between annotator A1 and annotator A2 was 0.7962, between annotator A1 and annotator A3 was 0.7796, and between annotator A2 and annotator A3 was 0.6213. Meanwhile, the average agreement among the three annotators based on Fleiss’ Kappa was 0.7323. Based on the standard interpretation of Kappa scores [11], these values indicate moderate to strong agreement, indicating a relatively high consistency among human annotators in assigning emotion categories.

**Table 8:** Inter-annotator agreement scores among three human annotators.

Pair	Score
A1 – A2 Cohen’s Kappa	0.7962
A1 – A3 Cohen’s Kappa	0.7796
A2 – A3 Cohen’s Kappa	0.6213
Human Annotator (A = 3) Fleiss’ Kappa	0.7323

### 4.5 Word embedding training

Two word embedding models, Word2Vec [6] and fastText [7], were trained using the EmoTweetID-Corpus.csv file. Both embedding models were created based on the skip-gram architecture [12] with a vector dimension of 300. Only tokens that appeared at least five times (with a context window size of 5) were included in the training data. The training was conducted for 10 epochs using the negative sampling strategy with five negative samples, without hierarchical softmax.

**Fig. 2:**
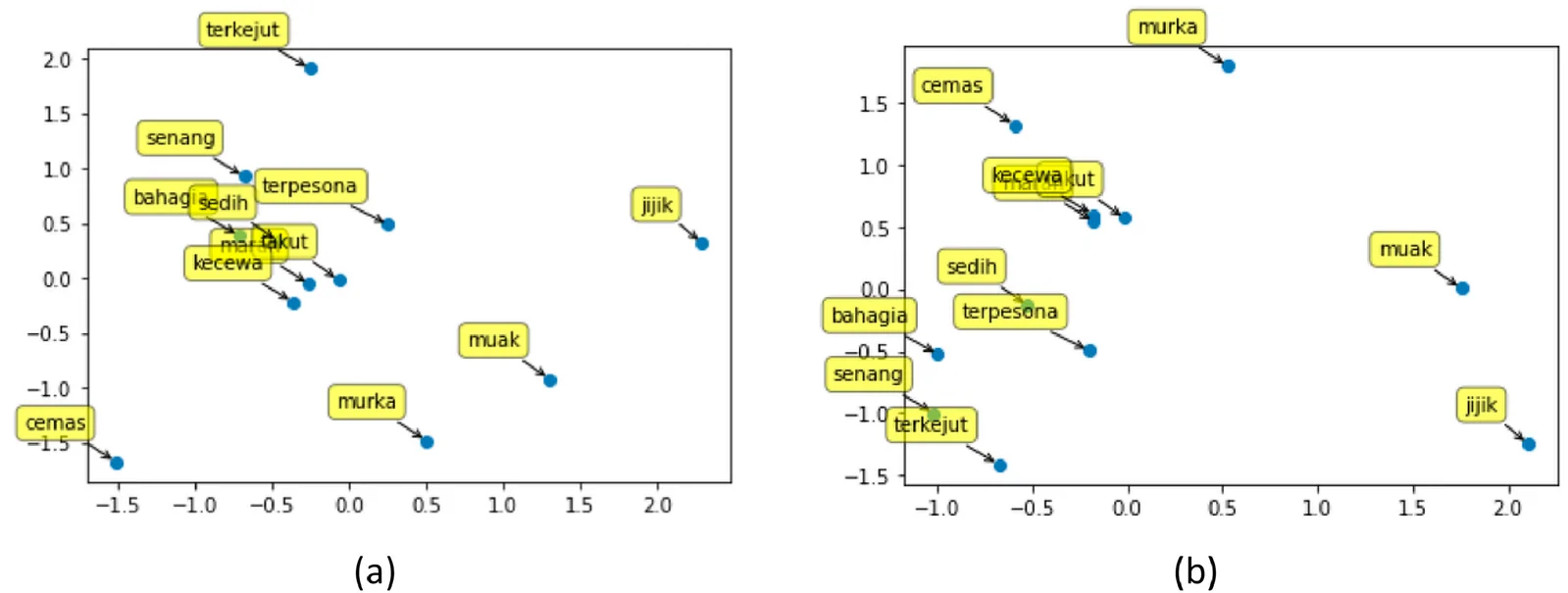
Two-dimensional visualization of pretrained word embeddings (a) Word2Vec and (b) fastText for the basic emotion keywords and their derived expressions, generated using models contained in TweetID-Word2Vec.zip and TweetID-fastText.zip.

The trained models were stored in both .npy and .wordvectors formats and compressed into .zip files compatible with the Gensim library (version 3.6.0 or later) [13]. These two pretrained word embedding models are publicly available in the Mendeley Data repository under the filenames TweetID-Word2Vec.zip and TweetID-fastText.zip. Fig. 2 shows the 2D visualization of the pretrained word embeddings of the basic emotion keywords and their derived expressions listed in Table 4. The complete training scripts for both Word2Vec and fastText, as well as the visualization script used to generate Fig. 2, are provided in the GitHub repository [9].

### 4.6 Baseline models for emotion classification

Two methods were applied to evaluate the initial performance of emotion classification on the EmoTweetID-Lexicon.csv and EmoTweetID-Human.csv datasets: traditional machine learning and deep learning. However, it should be noted that these baseline models are provided for reference and demonstration purposes only and are not intended to represent the state-of-the-art. The machine learning models used were the Decision Tree Classifier (DTC) with parameters min_samples_split=2 and min_samples_leaf=1, and the Support Vector Classifier (SVC) with a Radial Basis Function (RBF) kernel. Text representation was generated using Term Frequency-Inverse Document Frequency (TF-IDF) [14] with a combination of unigrams and bigrams, resulting in 39,389 features. Feature selection was performed using the chi-square method to reduce dimensionality, retaining the top 1,000 features with the highest association to the labels.

Bidirectional Long Short-Term Memory (BiLSTM) and Bidirectional Gated Recurrent Unit (BiGRU) architectures were implemented for the deep learning method. Both architectures included an embedding layer with a fixed dimension of 300, followed by either a BiLSTM or a BiGRU layer with 128 units (64 per direction). These were followed by a dense layer with 64 ReLU units, a dropout rate of 0.5, and a softmax output layer. Two types of text representation were employed for the deep learning methods. First, training without pretrained word embeddings, where tokens were converted into integer indices using a vocabulary restricted to the top 100 most frequent words (num_words = 100). Second, pretrained word embeddings from TweetID-Word2Vec.zip and TweetID-fastText.zip were used as text representation. All deep learning models were trained with consistent parameters: a batch size of 16, Adam optimizer with a learning rate of 2e-3, and 20 epochs with early stopping and a patience of 5.

The data were randomly split into 80 % for training and 20 % for testing using stratified sampling to preserve the proportion of emotion labels across both subsets. Model performance was evaluated using two primary metrics: accuracy and weighted F1-score [15]. Accuracy measures the overall correctness of predictions, while the weighted F1-score computes the weighted average of per-class precision and recall, with each class weighted proportionally to its support to account for label imbalance. A summary of the evaluation results across all models is presented in Table 9. Among all tested models and text representation methods, the BiLSTM with pretrained fastText embeddings achieved the highest performance on both lexicon-based and manually annotated datasets.

**Table 9:** Performance of baseline models for emotion classification using lexicon-based and human-annotated datasets.

Annotation	Model	Text representation	Accuracy	Weighted F1-score
Lexicon	DTC	TF-IDF (Uni-gram & Bi-gram)	0.6392	0.6403
	SVC	TF-IDF (Uni-gram & Bi-gram)	0.6125	0.6057
	BiLSTM	Tokenized-Padded Sequence	0.6582	0.6598
	BiLSTM	TweetID-Word2Vec	0.6776	0.6739
	**BiLSTM**	**TweetID-fastText**	**0.688**	**0.6894**
	BiGRU	Tokenized-Padded Sequence	0.6612	0.6603
	BiGRU	TweetID-Word2Vec	0.6686	0.671
	BiGRU	TweetID-fastText	0.6642	0.6615
Human	DTC	TF-IDF (Uni-gram & Bi-gram)	0.784	0.7838
	SVC	TF-IDF (Uni-gram & Bi-gram)	0.7639	0.7651
	BiLSTM	Tokenized-Padded Sequence	0.7875	0.7835
	BiLSTM	TweetID-Word2Vec	0.8172	0.8147
	**BiLSTM**	**TweetID-fastText**	**0.8291**	**0.8285**
	BiGRU	Tokenized-Padded Sequence	0.7979	0.7966
	BiGRU	TweetID-Word2Vec	0.8202	0.8195
	BiGRU	TweetID-fastText	0.8158	0.8145

## Limitations

The collected tweets are limited to those containing predefined keywords, introducing an inherent sampling bias that limits the dataset’s ability to represent the full spectrum of emotions, particularly tweets without explicit or implicit expressions, such as sarcasm and irony. The distribution of annotated emotion labels is imbalanced, potentially biasing the model toward the majority classes and reducing performance on downstream emotion classification tasks for minority emotion categories. Additionally, the data only cover tweets from 2020 to 2021, which may reflect the linguistic and emotional characteristics of that period, particularly during major global events. As language use and emotional expression on social media can evolve over time, the generalizability of the dataset to other time periods may be limited. Furthermore, the annotation scheme follows Ekman’s basic emotion model, which does not capture sentiment polarity dimensions like positive, negative, or neutral.

## Ethics Statement

Consent was obtained from the human annotators involved in this study. All collected tweets were anonymized, and data collection complied with Twitter’s Developer Agreement and Policy [16]. The dataset does not include raw tweets or identifiable user information to ensure privacy.

## CRediT Author Statement

**Kuncahyo Setyo Nugroho**: Conceptualization, Methodology, Software, Writing - Original Draft. **Fitra Abdurrachman Bachtiar**: Formal analysis, Investigation, Supervision. **Wayan Firdaus Mahmudy**: Validation, Supervision. **Matthew Martianus Henry**: Writing - Review & Editing. **Mahmud Isnan**: Writing - Review & Editing. **Gusti Pangestu**: Writing - Review & Editing. **Bens Pardamean**: Supervision.

## Data Availability

Mendeley DataEmoTweetID: Indonesian Emotion Tweet Dataset (Original data)
